# Optimization of renin-angiotensin-aldosterone inhibitor therapies for evidence-based indications: A call to action from the cardio-kidney community

**DOI:** 10.1016/j.ajpc.2024.100663

**Published:** 2025-06-09

**Authors:** Roberto Pecoits-Filho, Michelle M.Y. Wong, Monica Moorthy, Debasish Banerjee, Suman Behera, Viviane Calice-Silva, Mogamat-Yazied Chothia, Gates B. Colbert, Martha Gulati, Charles A. Herzog, Fadi Jouhra, Edgar V. Lerma, Charu Malik, Kershaw V. Patel, Amina Rakisheva, Giuseppe M.C. Rosano, Priyanka Satish, Henry H.L. Wu, Angela Yee-Moon Wang

**Affiliations:** a Arbor Research Collaborative for Health, Ann Arbor, MI, USA; b Pontificia Universidade Catolica do Parana, Curitiba, Brazil; c Division of Nephrology, Department of Medicine, University of British Columbia, Vancouver, British Columbia, Canada; d International Society of Nephrology, Brussels, Belgium; e Renal and Transplantation Unit, St George's University Hospitals NHS Foundation Trust, Molecular and Clinical Sciences Research Institute, St George's University of London, London, UK; f Division of Nephrology, William Osler Health System (Teaching Hospital, Michael G. DeGroote School of Medicine, McMaster University), Canada; g Research Department, Pro-Rim Foundation and School of Medicine, UNIVILLE, Joinville, Santa Catarina, Brazil; h Division of Nephrology, Department of Medicine, Stellenbosch University, Cape Town, South Africa; i Department of Nephrology, Texas A&M College of Medicine, Dallas, TX, USA; j Barbra Streisand Women's Heart Center, Department of Cardiology, Cedars Sinai Heart Institute, Los Angeles, CA, USA; k Cardiology Division, Department of Medicine, Hennepin Healthcare/University of Minnesota, Minneapolis, MN, USA; l Cardiology Department, St George's University Hospitals NHS Foundation Trust, Cardiology Clinical Academic Group, Molecular and Clinical Sciences Research Institute, St George's University of London, London, UK; m University of Illinois at Chicago/ Advocate Christ Medical Center, Oak Lawn, IL, USA; n Department of Cardiology, Houston Methodist DeBakey Heart & Vascular Center, Houston, TX, USA; o Cardiology Department, Scientific Research Institute of Cardiology and Internal Medicine, Almaty, Kazakhstan; p Centre for Clinical and Basic Research, IRCCS San Raffaele, Rome, Italy; q Renal Research, Kolling Institute of Medical Research, Royal North Shore Hospital & The University of Sydney, Sydney, Australia; r Department of Renal Medicine, Singapore General Hospital, Duke-National University of Singapore (NUS) Academic Medical Center, Singapore

**Keywords:** Renin angiotensin aldosterone inhibitors, Chronic kidney disease, Heart failure

Renin–angiotensin–aldosterone system inhibitor (RAASi) therapy, including angiotensin-converting enzyme inhibitors (ACEi), angiotensin receptor blockers (ARBs), angiotensin receptor-neprilysin inhibitors (ARNi), and steroidal and non-steroidal mineralocorticoid receptor antagonists (MRAs), is essential for treating diabetic and non-diabetic chronic kidney disease (CKD), heart failure (HF), and hypertension (HTN). However, these medications can lead to adverse events such as acute kidney injury and hyperkalemia. When patients experience these adverse events, RAASi therapy is often modified or stopped, potentially resulting in worse cardiovascular and kidney outcomes.

To manage patients effectively in this scenario, it is crucial to find a balance between the risks and benefits of RAASi therapy. This commentary reviews the evidence supporting the benefit/risk profile of RAASi in patients at the intersection of kidney and cardiovascular medicine, aiming to offer guidance for optimizing RAASi therapy in clinical practice. Additionally, a multi-stakeholder initiative is introduced to support healthcare professionals in implementing optimal RAASi therapy.

## 1 RAASi: a cornerstone therapy for the treatment of CKD (including diabetic), HF, and HTN

Given its strong evidence base, RAASi therapy is embedded in the guidelines for management of diabetes, CKD, HF, and HTN (Table 1) [[1], [2], [3], [4], [5]]. For patients with CKD, ACEi/ARB and non-steroidal MRA therapy decreases albuminuria, and risks of mortality, cardiovascular (CV) events, and progression to kidney failure (KF) [6,7]. Because the albuminuria-reducing effect is dose-related, guidelines recommend titration of ACEi or ARB to maximum approved doses or highest tolerated doses [1]. In patients with CKD and estimated glomerular filtration rate (eGFR) ≥30 ml/min/1.73 m^2^, steroidal MRAs in combination with ACEi or ARB reduce proteinuria and blood pressure, but there are uncertain effects on major CV and KF events due to limited data in trials [8]. The addition of the non-steroidal MRA, finerenone, on top of ACEi/ARB reduced the rate of both KF and CV events in patients with CKD with a satisfactory safety profile. In patients with HF with reduced ejection fraction (HFrEF), ACEi/ARB/ARNi, and steroidal MRAs have demonstrated benefits for mortality and HF hospitalization outcomes, and along with beta-blockers and sodium-glucose co-transporter 2 (SGLT-2) inhibitors, comprise the four pillars of guideline-directed medical therapy for HFrEF [3].

**Table 1:** Guideline recommendations for RAASi therapy[[1], [2], [3], [4], [5]].

Guideline	Recommendations/Practice points for treatment	Recommendation/Practice points for monitoring
KDIGO 2021 Clinical Practice Guideline for the Management of Blood Pressure in Chronic Kidney Disease[1] KDIGO 2022 Clinical Practice Guideline for Diabetes Management in Chronic Kidney Disease[2]	Recommend treatment with an ACEi or ARB be initiated in patients with diabetes, hypertension, and albuminuria, and that these medications be titrated to the highest approved dose that is tolerated (1B). Suggest a non-steroidal MRA with proven kidney or cardiovascular benefit for patients with T2D, eGFR ≥25 ml/min/1.73 m^2^, normal serum potassium concentration, and albuminuria (≥30 mg/g [≥3 mg/mmol]) despite maximum tolerated dose of RAASi (2A).	Monitor for changes in blood pressure, serum creatinine, and serum potassium within 2–4 weeks of initiation or increase in the dose of an ACEi or ARB. Continue ACEi or ARB therapy unless serum creatinine rises by more than 30 % within 4 weeks following initiation of treatment or an increase in dose.
2022 AHA/ACC/HFSA Guideline for the Management of Heart Failure[3]	In patients with HFrEF and NYHA class II to III symptoms, the use of ARNI is recommended to reduce morbidity and mortality (1A). In patients with previous or current symptoms of chronic HfrEF, the use of ACEi (or ARB if intolerant to ACEi because of cough or angioedema) is beneficial to reduce morbidity and mortality when the use of ARNI is not feasible (1A). In patients with LVEF ≤40 %, ACEi should be used to prevent symptomatic HF and reduce mortality (1A). In patients with a recent MI and LVEF ≤40 % who are intolerant to ACEi, ARB should be used to prevent symptomatic HF and reduce mortality (1B-R). In patients with HFrEF and NYHA class II to IV symptoms, an MRA (spironolactone or eplerenone) is recommended to reduce morbidity and mortality, if eGFR is >30 ml/mL/min/1.73 m^2^ and serum potassium <5.0 mEq/L (1A).	Careful monitoring of potassium, kidney function, and diuretic dosing should be performed at initiation and closely monitored thereafter to minimize risk of hyperkalemia and acute decline in kidney function. Regular checks of serum potassium and kidney function should be performed according to clinical status, approximately 1 week, then 4 weeks, then every 6 months after initiating or intensifying MRA, with more frequent testing as required clinically.
2021 ESC Guidelines for the diagnosis and treatment of acute and chronic heart failure[4]	An ACE-I is recommended for patients with HFrEF to reduce the risk of HF hospitalization and death (IA). An MRA is recommended for patients with HFrEF to reduce the risk of HF hospitalization and death (IA). RAASi should be uptitrated to the doses used in the clinical trials (or to maximally tolerated doses if that is not possible). Sacubitril/valsartan is recommended as a replacement for an ACEI in patients with HFrEF to reduce risk of HF hospitalization and death (IB). An ACE-I or ARB may be considered for patients with HFmrEF to reduce the risk of HF hospitalization and death (IIb C). An MRA may be considered for patients with HFmrEF to reduce the risk of HF hospitalization and death (IIb C).	*Re*-check blood chemistry (urea/BUN, creatinine, *K*+) 1–2 weeks after initiation and 1–2 weeks after final dose titration. Monitor blood chemistry 4-monthly thereafter.
2017 ACC/AHA/AAPA/ABC/ACPM/AGS/APhA/ASH/ASPC/NMA/ PCNA Guideline for the prevention, detection, evaluation and management of high blood pressure in adults[5]	Adults with HFpEF and persistent hypertension after management of volume overload should be prescribed ACE inhibitors or ARBs and beta blockers titrated to attain SBP of less than 130 mm Hg (1 C-LD). In adults with hypertension and CKD (stage 3 or higher or stage 1 or 2 with albuminuria [≥300 mg/d, or ≥300 mg/g albumin-to-creatinine ratio or the equivalent in the first morning void]), treatment with an ACE inhibitor is reasonable to slow kidney disease progression (IIa B-R). In adults with hypertension and CKD (stage 3 or higher or stage 1 or 2 with albuminuria [≥300 mg/d, or ≥300 mg/g albumin-to-creatinine ratio in the first morning void]), treatment with an ARB may be reasonable if an ACE inhibitor is not tolerated (IIb C-EO). In adults with DM and hypertension, ACE inhibitors or ARBs may be considered in the presence of albuminuria (IIb B-NR).	

Abbreviations: ACEi, ACE-I, and ACEI, angiotensin-converting enzyme inhibitors; ARB, angiotensin receptor blockers; ARNI, angiotensin receptor-neprilysin inhibitors; AHA/ACC/HFSA, American Heart Association/American College of Cardiology/Heart Failure Society of America; AAPA/ABC/ACPM/AGS/APhA/ASH/ASPC/NMA/ PCNA, American Academy of Physician Assistants/ Association of Black Cardiologists/American College of Preventive Medicine/American Geriatrics Society/American Pharmacists Association/American Society of Hypertension/American Society for Preventive Cardiology/National Medical Association/Preventive Cardiovascular Nurses Association; BUN, blood urea nitrogen; CKD, chronic kidney disease; DM, diabetes mellitus; eGFR, estimated glomerular filtration rate; ESC, European Society of Cardiology; HF, heart failure; HFmrEF, heart failure with mildly reduced ejection fraction; HFpEF, heart failure with preserved ejection fraction; HFrEF, heart failure with reduced ejection fraction; HTN, hypertension; *K*+, potassium; KDIGO, Kidney Disease: Improving Global Outcomes; LVEF, left ventricular ejection fraction; MI, myocardial infarction; MRA, mineralocorticoid receptor antagonists; NYHA, New York Heart Association; RAASi, renin–angiotensin–aldosterone system inhibitor; SBP, systolic blood pressure; T2D, type 2 diabetes.

If applicable, class of recommendation and level of evidence are listed in parentheses.

## 2 Underutilization of RAASi in evidence-based indication: pitfalls and need for action

In everyday practice, the dosing and maintenance of RAASi is suboptimal, with only approximately 25–45 % of patients reaching target dosing [9]. Additionally more than 10 % of patients may have their RAASi therapy discontinued altogether, particularly those at the lower range of eGFR. In the specific setting of HF, 27 %, and 67 % were not prescribed ACEi/ARB/ARNi, and MRA therapy, respectively [10]. MRAs are notoriously underutilized in patients with HFrEF, with studies showing an overall adherence rate of only approximately 50 % due to hyperkalemia risk [11,12]. In CKD, though prescription patterns vary by country, there is general underuse of RAASi [13]. Among patients with both HFrEF and CKD, RAASi prescription was lowest at eGFR <30 ml/min/1.73 m^2^, particularly for ACEi/ARB [14]. Furthermore, given the common interface between HFrEF and CKD, RAASi therapies are often used in patients with at least some degree of kidney dysfunction. In randomized controlled trials for HF, approximately 33 % of enrolled patients had an eGFR between 60 - 90 ml/min/1.73 m^2^, and 30–35 % of patients had eGFR below 60 ml/min/1.73 m^2^ [15].

In a real-world study of US patients with albuminuria prescribed ACEi or ARB, demographic features associated with lower odds of maximal ACEi/ARB dosing included younger age (<40 years), female sex, and Hispanic ethnicity [16].

A small observational study [17] showed that RAASi use in patients with advanced CKD may accelerate the need for kidney replacement therapy (KRT), providing the rationale for discontinuing RAASi below a certain (i.e., 20 ml/min/1.73 m^2^) eGFR threshold. However, recent evidence from a small randomized controlled trial demonstrated no adverse effects of RAASi on the risk of progression to KRT or hyperkalemia in patients with eGFR below 30 ml/min/1.73 m^2^ [18]. There is also growing evidence that stopping RAASi may increase risk of CV events and mortality, including in patients with advanced CKD [19]. Moreover, studies have demonstrated that sub-optimal RAASi therapy is associated with greater risk of mortality and CV adverse events in CKD and HF populations [20,21]. The current CKD and HF guidelines recommend that discontinuation of RAASi in evidence-based indications should be the last resort after failing to manage and prevent hyperkalemia adverse-effects of these life-saving therapies [22]. Dissemination of these concepts and recommendations are crucial for improving clinical outcomes in these conditions.

## 3 Reasons for suboptimal utilization of RAASi in evidence-based indications: hyperkalemia and acute declines in kidney function

Non-adherence to RAASi therapy has been largely attributed to hyperkalemia and acute worsening in kidney function. In both inpatient and outpatient settings, studies have shown that 10–38 % of hospitalized patients on RAASi therapy developed hyperkalemia during hospitalization and 10 % of patients developed severe hyperkalemia (serum potassium >6.0 mmol/L) within 1 year of follow-up [23,24]. The prevalence of hyperkalemia in advanced CKD is up to 73 % [25]. In the setting of chronic HF, hyperkalemia occurs in up to 40 % of patients [26]. It's estimated that 5–25 % of patients with CKD develop hyperkalemia after starting RAASi [27]. In patients with resistant hypertension, combination therapy with multiple agents that can raise serum potassium increases the risk of hyperkalemia, and some etiologies of resistant hypertension, such as renovascular hypertension, may increase the risk of hyperkalemia and cause acute decline in kidney function shortly after initiation of RAASi therapy. In addition to renal vascular disease, other causes of acute declines in kidney function following RAASi initiation are low mean arterial pressure, commonly observed with HF or hypotension, and volume depletion [28].

Hyperkalemia occurred more frequently in patients treated with steroidal and non-steroidal MRAs vs. placebo in clinical trial settings, a comparative analysis suggests finenerone at 5–10 mg daily is associated with a lower incidence of hyperkalemia compared with spironolactone 25–50 mg daily [29]. However, prospective head-to-head RCTs or real-world studies are needed to clarify the safety profiles of steroidal vs*.* non-steroidal MRAs.

### 3.1 Monitoring hyperkalemia and acute declines in kidney function in the context of RAASi optimization

Hyperkalemia and transient renal declines are known concerns with RAASi therapy especially among people with advanced age and greater number of comorbidities, but evidence shows these risks are usually mild, transient, and manageable. . The change in serum potassium with initiation of ACEi or ARB is typically within +0.5 mmol/L in patients with CKD and HTN [30]. In CKD, guidelines recommend that RAASi (including non-steroidal-MRAs) should not be initiated when serum potassium is >5.0 mmol/L, and to monitor serum creatinine and potassium after starting or changing dose (Table 1) [1,26]. Rather than limiting therapy, proactive strategies—routine monitoring, diuretics, dietary guidance, and potassium binders—enable safe continuation. Since real-world practice often deviates from guidelines due to safety concerns, emphasizing structured monitoring and mitigation is key to overcoming therapeutic inertia and maximizing RAASi benefits.

### 3.2 Managing hyperkalemia and acute declines in kidney function in the context of RAASi optimization

Given the potential risks of hyperkalemia and acute declines in kidney function with RAASi, there is a growing discrepancy between current guidelines and real-world practice, and an increasing need to prevent or improve management of these adverse events once developed.

The European Society of Cardiology (ESC) recommended multiple strategies for managing hyperkalemia, including diuretics, eliminating non-essential hyperkalemia-inducing drugs (i.e., NSAIDs), dietary modifications [26] and the use of potassium binders if available. A Kidney Disease: Improving Global Outcomes (KDIGO) consensus meeting emphasized these preferred measures to RAASi discontinuation/dose reduction, a concept that has impacted the recent revision of guidelines for blood pressure and diabetes management in CKD, in which a “*stop/reduce dose of RAASi as a last resort*” approach is recommended [1,2]. Additionally, the utilization of potassium binders in combination with RAASi therapy can help to optimize treatment and improve patient outcomes by reducing the risk of hyperkalemia while maintaining the benefits of RAASi therapy. Currently, the use of sodium/calcium polystyrene sulphonate (SPS/CPS) for management of hyperkalemia is low and chronic use is not recommended due to gastrointestinal adverse effects ranging from nausea and constipation to intestinal obstruction and colonic ischemia/necrosis [31,32]. Novel potassium binders, including patiromer and sodium zirconium cyclosilicate, are better tolerated and can facilitate optimization of RAASi among patients with persistent hyperkalemia[33]; the overall evidence that this strategy can improve outcomes is still uncertain but evolving (currently a class 2b, level B-R recommendation in 2022 AHA/ACC/HFSA Heart Failure Guideline) [3].

## 4 A multi-stakeholder initiative: development of a toolkit to improve implementation of RAASi therapy

Many barriers to implementing guideline-based cardio-renal-metabolic care exist in real-word settings. Clinical inertia is related to system, patient, and physician factors [34]. Patient non-adherence to treatment may stem from cost, cultural/personal attitudes regarding treatments, and poor health literacy [35]. In a HF study, physician non-adherence to guidelines was greater among patients with more comorbidities, greater severity of HF, and ethnic minority patients. Underlying causes include provider bias/prejudice, time pressure, and clinical uncertainty, and lack of training focused on therapeutic goals [16,35,36]. To address physician factors in GDMT in chronic HF, previous implementation studies have assessed the effectiveness of electronic-health-record alerts to providers in pragmatic randomized controlled trials. Providing alerts with individualized guidance on GDMT during office visits demonstrated a significant increase in the number of GDMT classes prescribed [37].

The International Society of Nephrology (ISN) convened experts from the American Society for Preventive Cardiology (ASPC), the Heart Failure Association of the ESC (ESC—HFA), Kidney Disease: Improving Global Outcomes (KDIGO), and the Renal Physicians Association (RPA), to enhance evidence-based therapies at the intersection of CV and kidney medicine. The development of this toolkit was the first activity of this group, who aims to address other unmet needs in the area.

**Fig. 1:**
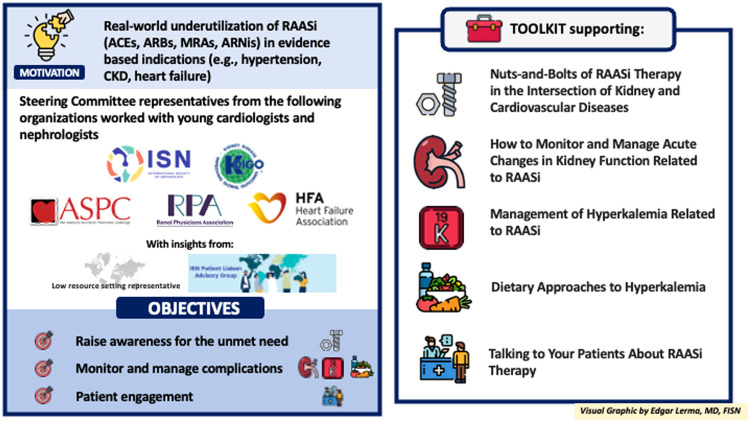
Optimization of RAASi Therapy Toolkit ACEs = angiotensin-converting enzyme inhibitor/s ARBs = angiotensin receptor blocker/s ARNis= angiotensin receptor-neprilysin inhibitor/s CKD = chronic kidney disease MRA = non-steroidal mineralocorticoid receptor antagonist/s RAASi = renin–angiotensin–aldosterone system inhibitor.

Five educational and clinical tools were created to improve adherence to guidelines and elevate patient care quality, covering important aspects of RAASi therapy. “The nuts-and-bolts of RAASi therapy” tool describes therapy basics (Fig. 1). The "monitor and manage hyperkalemia related to RAASi" tool addresses multifactorial causes of hyperkalemia. The "dietary approaches to hyperkalemia" tool promotes a balanced diet over extreme restrictions and dispels potassium-related myths. The "monitor and manage acute changes in kidney function related to RAASi" tool outlines creatinine monitoring recommendations and action thresholds. The "talking to your patients about RAASi therapy" tool supports clinicians in counseling patients starting RAASi, emphasizing a patient-centered approach. These tools are available online at: https://www.theisn.org/initiatives/toolkits/raasi-toolkit/.While a toolkit is integral to an educational intervention, it is just one component of an implementation study, and other factors, including the context of a particular setting and the method of delivery of the intervention, must be considered. The ‘Optimization of RAASi’ toolkit can be implemented into clinical settings using electronic or paper copies as handouts/posters, or embedding the toolkit link within the EMR or placing macros in clinical documentation to facilitate communication between health care providers. To optimize comprehensive medication management. multidisciplinary care teams, including clinical pharmacists, can utilize the toolkit during clinic visits. A multifaceted approach combining the toolkit with active strategies such as educational workshops, webinars, and audit/feedback on prescribing practices can maximize impact [38]. A series of ISN-led webinars is currently ongoing. Future implementation studies can evaluate both implementation- and effectiveness outcomes of multipronged strategies that include the toolkit.

In summary, the endorsed tools developed through this cross-specialty collaboration with scientific societies prioritize a patient-centered approach. The project's uniqueness lies in the diverse composition of the team, including experts from various geographies and backgrounds, and the incorporation of patients' voices. By aligning with operational workflows and adopting a pragmatic approach, global implementation of these valuable resources is facilitated. Healthcare professionals can access them at: www.theisn.org/initiatives/toolkits/raasi-toolkit/. Embracing these evidence-based strategies will undoubtedly advance patient care in CV and kidney medicine.

## Author declaration

We wish to confirm that there are no known conflicts of interest associated with this publication and there has been no significant financial support for this work that could have influenced its outcome.

Other potential conflicts of interest unrelated to this work are displayed in individual forms, as well as listed in the disclosures section of the manuscript.

The International Society of Nephrology (ISN) led this work. This work was supported by an unrestricted educational grant from AstraZeneca and CSL Vifor. The authors did not receive any financial support to work on this project.

We confirm that the manuscript has been read and approved by all named authors and that there are no other persons who satisfied the criteria for authorship but are not listed. We further confirm that the order of authors listed in the manuscript has been approved by all of us.

We confirm that we have given due consideration to the protection of intellectual property associated with this work and that there are no impediments to publication, including the timing of publication, with respect to intellectual property. In so doing we confirm that we have followed the regulations of our institutions concerning intellectual property.

We understand that the Corresponding Author is the sole contact for the Editorial process (including Editorial Manager and direct communications with the office). He/she is responsible for communicating with the other authors about progress, submissions of revisions and final approval of proofs. We confirm that we have provided a current, correct email address which is accessible by the Corresponding Author.

## Disclosures

RPF reports non-financial support from Fresenius Medical Care, Bayer, Astra Zeneca, Novo Nordisk, Fibrigen, Akebia, Boehringer, personal fees from Geroge Clinical, outside the submitted work; and RPF is employed by Arbor Research Collaborative for Health, who runs the DOPPS studies. Global support for the ongoing DOPPS Programs is provided without restriction on publications by a variety of funders. Funding is provided to Arbor Research Collaborative for Health and not to RPF directly. https://www.dopps.org/AboutUs/Support.aspx.

MMYW reports personal fees from George Clinical (consultant), personal fees from Astra Zeneca (Advisory Board on CKD early identification and intervention in Primary Care), and grants from Michael Smith Health Research BC (Research salary support), outside the submitted work.

MM reports personal fees from The International Society of Nephrology (consultant) outside the submitted work.

DB reports grants from AstraZeneca, BHF, and KRUK[JD1], and personal fees from AstraZeneca, Bayer, and Vifor Pharma, outside the submitted work.

GBC reports personal fees from AstraZeneca and Bayer, outside the submitted work.

CAH reports equity (stock) of Boston Scientific, Johnson &Johnson, Merck, and Pfizer, grants from Relypsa/Vifor and Bristol-Meyers Squibb, and personal fees from AstraZeneca, Bayer, Diamedica, Fibrogen, Merck, NxStage, and Relypsa/Vifor, outside the submitted work.

EVL reports personal fees from Akebia/Otsuka, Astra Zeneca, Bayer, GSK, Otsuka, Travere, Vertex, Vifor, Elsevier, McGraw-Hill, Springer, and Wolters Kluwer, outside the submitted work.

CM reports personal fees from The International Society of Nephrology (employee), outside the submitted work.

AW Received speaker fees from Astra Zeneca, Bayer AG and Fresenius Kabi Received Travel fees from Astra Zeneca, Bayer AG and Fresenius Kabi Served as advisory board member of Fresenius Kabi Served as Executive Committee member of CSL Behring sponsored trial.

All other authors have nothing to disclose.

## Subject area

Chronic kidney disease, heart failure

## Funding

The International Society of Nephrology (ISN) led this work. This work was supported by an unrestricted educational grant from AstraZeneca and CSL Vifor.

## CRediT authorship contribution statement

**Roberto Pecoits-Filho:** Writing – review & editing, Writing – original draft. **Michelle M.Y. Wong:** Writing – review & editing, Writing – original draft. **Monica Moorthy:** Writing – review & editing. **Debasish Banerjee:** Writing – review & editing. **Suman Behera:** Writing – review & editing. **Viviane Calice-Silva:** Writing – review & editing. **Mogamat-Yazied Chothia:** Writing – review & editing. **Gates B. Colbert:** Writing – review & editing. **Martha Gulati:** Writing – review & editing. **Charles A. Herzog:** Writing – review & editing. **Fadi Jouhra:** Writing – review & editing. **Edgar V. Lerma:** Writing – review & editing. **Charu Malik:** Writing – review & editing. **Kershaw V. Patel:** Writing – review & editing. **Amina Rakisheva:** Writing – review & editing. **Giuseppe M.C. Rosano:** Writing – review & editing. **Priyanka Satish:** Writing – review & editing. **Henry H.L. Wu:** Writing – review & editing. **Angela Yee-Moon Wang:** Writing – review & editing.

## Declaration of competing interest

We wish to confirm that there are no known conflicts of interest associated with this publication and there has been no significant financial support for this work that could have influenced its outcome.
